# Digital technology for health sector governance in low and middle income countries: a scoping review

**DOI:** 10.7189/jogh.06.020408

**Published:** 2016-12

**Authors:** Isaac Holeman, Tara Patricia Cookson, Claudia Pagliari

**Affiliations:** 1 Cambridge Judge Business School, Cambridge, UK; 2 Medic Mobile, San Francisco, CA, USA; 3 Edinburgh Global Health Academy & Usher Institute of Population Health Sciences and Informatics, University of Edinburgh, Edinburgh, Scotland, UK; 4 Department of Geography, University of Cambridge, Cambridge, UK

## Abstract

**Background:**

Poor governance impedes the provision of equitable and cost–effective health care in many low– and middle–income countries (LMICs). Although systemic problems such as corruption and inefficiency have been characterized as intractable, “good governance” interventions that promote transparency, accountability and public participation have yielded encouraging results. Mobile phones and other Information and Communication Technologies (ICTs) are beginning to play a role in these interventions, but little is known about their use and effects in the context of LMIC health care.

**Methods:**

Multi–stage scoping review: Research questions and scope were refined through a landscape scan of relevant implementation activities and by analyzing related concepts in the literature. Relevant studies were identified through iterative Internet searches (Google, Google Scholar), a systematic search of academic databases (PubMed, Web of Science), social media crowdsourcing (targeted LinkedIn and Twitter appeals) and reading reference lists and websites of relevant organizations. Parallel expert interviews helped to verify concepts and emerging findings and identified additional studies for inclusion. Results were charted, analyzed thematically and summarized.

**Results:**

We identified 34 articles from a wide range of disciplines and sectors, including 17 published research articles and 17 grey literature reports. Analysis of these articles revealed 15 distinct ways of using ICTs for good governance activities in LMIC health care. These use cases clustered into four conceptual categories: 1) gathering and verifying information on services to improve transparency and auditability 2) aggregating and visualizing data to aid communication and decision making 3) mobilizing citizens in reporting poor practices to improve accountability and quality and 4) automating and auditing processes to prevent fraud. Despite a considerable amount of implementation activity, we identified little formal evaluative research.

**Conclusion:**

Innovative digital approaches are increasingly being used to facilitate good governance in the health sectors of LMICs but evidence of their effectiveness is still limited. More empirical studies are needed to measure concrete impacts, document mechanisms of action, and elucidate the political and sociotechnical dynamics that make designing and implementing ICTs for good governance so complex. Many digital good governance interventions are driven by an assumption that transparency alone will effect change; however responsive feedback mechanisms are also likely to be necessary.

Worldwide, poor health sector governance results in inefficiency, waste, error and fraud, compromising the integrity of health services and the equitable delivery of patient care. The problem is particularly acute in low– and middle–income countries (LMICs), where corruption in medicine has been referred to as an “open secret” [1]. Observing that developing countries lose some US$ 1.26 trillion per year to corruption, bribery, theft and tax evasion, the United Nations’ Sustainable Development Goal 16 calls for more transparent, accountable and participatory institutions at all levels of government [2]. The concept of “good governance” embeds these objectives within an overarching ethic of “responsible use of power at all levels of institutions” [3].

The complex organizational, political and socio–cultural dynamics associated with poor governance can seem intractable, but discrete and replicable interventions for tackling these problems have yielded encouraging results. For example, a randomized trial of Community Score Cards in Uganda was associated with substantial decreases in provider absenteeism and wait times, a 20% rise in outpatient service utilization, and a 33% reduction in child mortality in just one year, at a cost of only US$ 3 per household [4].

With the spread of the Internet, mobile phones and social media, approaches toward encouraging good governance are taking new digital forms. Some are emerging organically through social movements aimed at effecting change through group pressure, while others have been intentionally designed to enable citizens or co–workers to report poor practices directly to health organizations or to an oversight body. Within the global health and development community such approaches are becoming well established if not yet widespread. For example, the anti–corruption platform ipaidabribe.com, developed by the not–for–profit organization Janaagraha, is now widely used across India [5] and UNICEF’s community empowerment platform U–Report boasts millions of users worldwide [6].

Reviewing such interventions is challenging because researchers use “governance” and related terms in various ways, as outlined in **Table 1**. This report focuses on uses of digital technology for “good governance”; promoting responsive, participatory, transparent, accountable, equitable and effective institutions [3]. Our review includes bottom–up (citizen–driven) transparency and accountability initiatives [16–19], top–down e–Governance (and e–democracy) projects [7], and public–private partnerships that build or collaboratively implement governance–enabling technologies [20]. By “interventions” we mean potentially replicable projects, programs or social innovations that address dishonest or corrupt practices on the part of health care practitioners and leaders, or that foster citizen participation and make governance more responsive to health care consumers. These interventions address concerns as diverse as public participation, corruption, whistleblowing, bribery, fraud, theft, absenteeism, harassment, discrimination and unfair allocation of funding or government contracts. By “digital” we are referring to mobile ICT, social media and other digital innovations that may support these “good governance” objectives, rather than broader eHealth infrastructure such as electronic health record systems.

**Table 1:** Differentiating “good–governance” from eGovernment and related terms*

ICT for good governance	We use the term “ICTs for good governance” for interventions that involve ICTs, that are aligned with Sustainable Development Goal 16’s call for more transparent, accountable and participatory institutions [2], and that are concerned with “responsible use of power at all levels of institutions” [3]. This includes initiatives that are bottom–up, top–down, or include elements of both through public private partnerships. eGovernance is an overlapping term; all interventions in our review involved ICTs and many also involved non–digital elements.
eGovernment	The term eGovernment refers broadly to the digitization of government services, often with a technical orientation toward improving efficiency or quality of services rather than the responsible exercise of power [7]. While many sources use eGovernment and governance more or less interchangeably, our review focused exclusively on replicable governance interventions that targeted practical, concrete and measurable concerns with government performance by promoting responsible exercise of power.
Governance of eHealth / Health Information Governance	This literature has its origins in the large–scale implementation of information systems in health care, more recently including the use of mHealth and personal digital health devices. The storage, use and sharing of personal data in these new environments raises risks for information security and privacy, which have technological, legal/regulatory and ethical/societal implications. The word governance is often used to describe the policies and processes of oversight required to ensure the security and trustworthiness of such systems. It may also be used to refer to the management structures involved in collective oversight of eHealth initiatives.
	Governance of health systems through information is another theme in this literature, concerning the best use of data for supporting health care planning, coordination, quality improvement and evaluation, in common with the “Learning Health Systems” concept [8].
Clinical governance	This term underscores continuous improvement of health care service quality [9], generally through organizational integration of financial, performance and clinical quality [10].
Participatory governance	This approach to governance emphasizes the strengthening of citizen voices, and particularly those of marginalized groups, in decision–making processes. Processes of deliberation, consultation and mobilization are particularly relevant [11].
Global governance	This literature takes a macro perspective in studying worldwide governance of contemporary health issues. For instance, it is concerned with the role of international organizations in assisting countries to manage cross–border risks to public health security and support improvement of health outcomes [12].
	Recent work in this global governance vein has addressed the challenge of achieving the goal of “health for all by the year 2000” in a free market economy [13], the proliferation of global health NGOs and the potential of the World Health Organization as a coordinating and governing body [14], and structural governance challenges related to national sovereignty or the accountability of non–state actors [15].

ICT – Information and communication technologies, NGO – non-governmental organization

*Includes terms most closely related to the review topic and not others such as corporate governance, which concerns companies.

While relevant reviews exist in the contexts of sustainable development [21], and health information governance [22], to the best of our knowledge there has been no systematic overview of how ICTs are being used to increase the transparency, accountability or trustworthiness of health care providers, organizations and the public health sector as a whole in LMICs. The report summarized in this paper set out to map and describe the existing landscape of digital good–governance interventions for strengthening health systems in LMICs, and to highlight opportunities for future research and innovation [23].

## METHODS

### Study design

We undertook a phased scoping review including a landscape scan of implementation activities and a systematic keyword search of academic databases, guided by interviews with experts and practitioners in the field and an emergent theoretical framework. The scoping review methodology is increasingly used for mapping areas that are nascent or widely scattered [24], where conventional searches of academic databases are less likely to be fruitful. This approach can be used to understand key concepts, theories and sources of evidence as a means of guiding new innovations, empirical research or systematic reviews, and informing policymakers. Scoping reviews typically do not involve critical appraisal of study methodology or detailed extraction of outcomes data, since they are chiefly concerned with mapping the landscape of evidence rather than establishing the effectiveness of particular interventions [24,25]. **Table 2** summarizes the differences between scoping reviews and comprehensive systematic reviews.

**Table 2:** Differences between comprehensive systematic reviews and scoping reviews*

Comprehensive Systematic Review	Scoping Review
Focused research question with narrow parameters	Research question(s) often broad
Inclusion/exclusion defined at outset	Inclusion/exclusion developed post hoc
Study quality filters applied	Study quality not an initial priority
Detailed data extraction	May or may not involve data extraction
Quantitative synthesis often performed	Synthesis more likely to be qualitative/thematic
Formally assess the quality of studies and generate a conclusion relating to focused research question	Used to map the landscape of peer–reviewed research and gray literature, identify gaps and opportunities

*Based on a Cochrane update by Armstrong et al [24].

As outlined in **Box 1**, we performed all of the activities recommended in Arksey and O’Malley’s widely cited scoping review framework [25], as well as a landscape scan of implementation activities and consultation with experts, which are typically regarded as optional. We summarize each of these phases below and discuss our methodology more exhaustively in the complete version of the report on which this article is based [26].

Box 1 **Scoping stages used in this review**

1. Refine research questions by reviewing the literature on relevant theoretical concepts

2. Undertake landscape scan of implementation activity: Identify key actors, project reports and gray literature relevant to digital technology and some aspect of good governance

3. Based on 1 and 2, define a strategy for systematically searching databases of peer–reviewed research

4. Apply agreed inclusion and exclusion criteria to select relevant studies

5. Converge results of database searches with products of snowball sampling from landscape scan

6. Chart and summarize the data

7. Consult with key experts to elaborate concepts and identify other research

8. Collate, summarize and report the results

### Mapping concepts and refining the research questions

The project began with a broad remit to review the evidence on innovative uses of mobile technology for strengthening “leadership, management and governance” in the health sectors of low– and middle–income countries in line with the topic areas of the funding scheme. In order to better refine the scope and focus, and avoid duplication, we began by examining existing reviews and commentary in the field, to differentiate the above three sub–topics and determine where the important knowledge gaps lie. This revealed an important gap in the literature concerning uses of digital technology for health sector governance, in contrast to a more extensive literature related to health care management and leadership issues, specifically the “good governance” agenda described in our introduction and in **Table 1**.

### Landscape scan of implementation activity

Based on the above, and informal discussions with experts known to our team, we determined that ICT for health governance is an active area of applied activity, although somewhat under–researched. For this reason, we began by seeking case reports to better understand the nature of projects in this area, beginning with those we were familiar with and snowballing via web links, tracing the work of key organizations and funding streams, and undertaking targeted keyword searches in Google and Google Scholar. Case reports included “grey literature” such as project reports, compendia of mHealth/eHealth initiatives, and websites and blog posts describing active or completed projects. Searches at this early stage were conducted in English, Spanish and Portuguese; since members of the team are fluent in these languages. From an initially large and diffuse set of results we identified 22 case reports that reflected the review’s iteratively refined focus on good governance, rather than management or leadership. We also developed a list of key actors who surfaced repeatedly in relevant case reports, including funders (eg, US government, Swedish government), research organizations (eg, the Anti–Corruption Resource Centre, Transparency International) and technology organizations (eg, Ushahidi) [26]. In a spreadsheet we summarized each report’s “use case” and key themes. Based on this spreadsheet we continued to iteratively refine our search terms and initiate the search for peer–reviewed literature. Our approach reflects similar landscape scans undertaken as part of other scoping review exercises [27,28].

### Systematically searching academic databases and applying inclusion criteria

Based on the initial concept mapping exercise and landscape scan, we defined a strategy for systematically searching for articles published in English and indexed in PubMed (for medical literature) or Web of Science (for interdisciplinary literature). Searches included combinations of the following terms: “governance,” “transparency,” “accountability,” “participation,” “participatory,” “stakeholder engagement,” “corruption,” “absenteeism,” “mHealth,” “eHealth”, “mobile phone”, “social media” and “digital.” Further articles were identified by examining reference lists and through key informant interviews.

**Figure 1:**
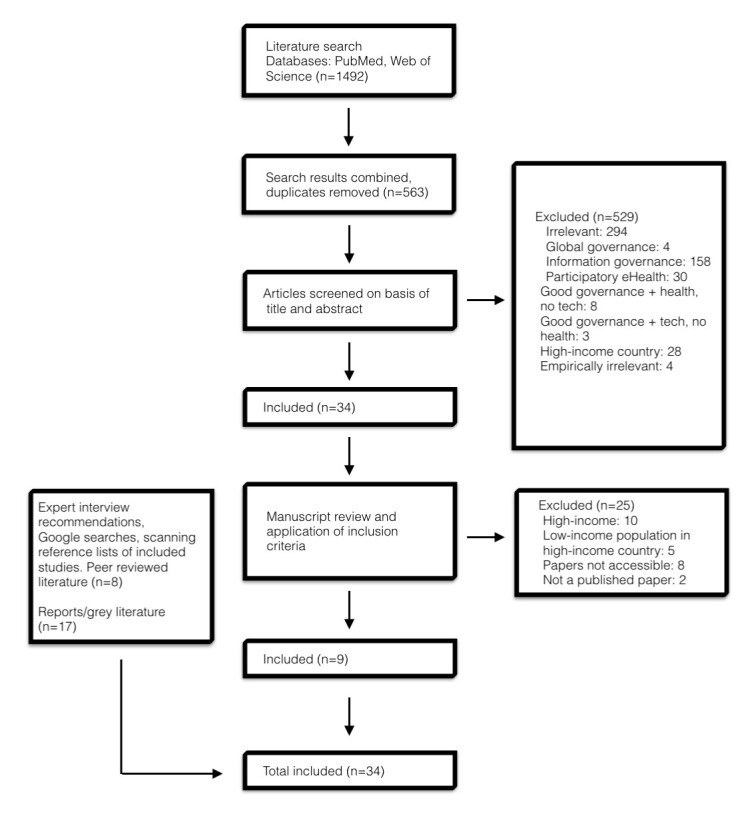
PRISMA flowchart illustrating the search process.

To be eligible for inclusion articles had to describe *digital technology* for *good governance* purposes in the *health sector* of a *low or middle–income country*. Those that did not encompass all four features, or were purely concerned with information governance or project management in the context of an mHealth or eHealth project, were excluded. **Figure 1** shows a PRISMA diagram representing the formal literature review and sifting process.

### Charting and analyzing the data

Due to resource constraints, articles that could not be accessed through the University of Cambridge or University of Edinburgh e–libraries were excluded. The remaining articles were downloaded for full review. In keeping with standard scoping review frameworks, we charted these studies according to key themes rather than performing full data extraction. We also followed Levac et al.’s [29] recommendation to make charting an iterative process by continually updating the data–charting spread sheet to fit the study data being extracted. The fields used for data compilation and analysis were as follows:

1. Author(s), year of publication, study location
2. Study type/methodology
3. Problem(s) the program aimed to address
4. Technology used
5. Intervention use cases (eg, data collection with mobile apps, interactive digital mapping) and categories (eg, information gathering, mobilization).

### Consultation with expert practitioners and researchers

To validate and develop our emerging insights, we posted questions to relevant ICT and global health–oriented email lists and online forums, including GHDonline, the mHealth Working Group listserv, and several LinkedIn groups. Through these posts we identified a number of additional gray literature reports and peer–reviewed articles. Key respondent interviews were also undertaken as a means of identifying additional unpublished work, testing emergent themes, informing iterative improvements to the analysis, and supporting interpretation with reference to “real world” challenges. Interview participants were 10 purposively sampled practitioners and researchers affiliated with key organizations or technology projects that emerged repeatedly in the searches, including men and women with work experience in Africa, Asia and Latin America. Interviews were informal and unstructured, lasting for approximately 45 minutes each.

## RESULTS

PubMed and Web of Science searches yielded 1492 results, of which nine met all the inclusion criteria (n = 9). Expert interviews, social media recommendations, Google searches and analyses of websites and reference lists yielded 25 additional papers, including peer–reviewed articles (n = 8), and technical reports/gray literature (n = 17). In total thirty–four published research articles (n = 17) and reports (n = 17) were included (Appendices S1 and S2 in **Online Supplementary Document(Online Supplementary Document)**).

### Composition of the evidence–base

Peer–reviewed evaluative research was sparse relative to other article types. The majority of included articles were identified through iterative and adaptive online searches (n = 25) rather than using keywords to systematically search academic databases (n = 9). This reflects the fact that academic articles used different terminologies and came from disparate communities of practice, including political science, sociology and medicine, confirming the appropriateness of our iterative scoping methodology. The technical reports came from WHO, the World Bank, or non–governmental organizations. Most of the peer–reviewed articles and technical reports included conceptual frameworks or descriptive case examples, rather than evaluative research.

### Common uses of ICT for Good Governance Interventions in the health sector

**Figure 2:**
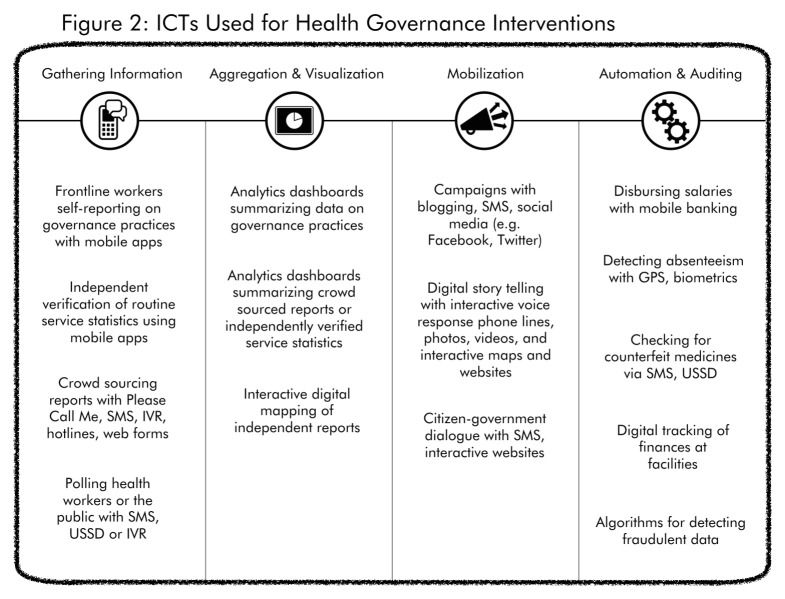
Information and communication technologies (ICTs) used for health governance interventions.

Our analysis revealed 15 distinct ways of using ICTs as components of health governance interventions, or use cases. We grouped these into four conceptual categories: 1) gathering and verifying information on health services to improve transparency and auditability, 2) aggregating and visualizing data to aid communication and decision making, 3) mobilizing citizens in reporting poor practices to improve accountability and quality, and 4) automating and auditing processes to address fraud or similar inappropriate practices. **Figure 2** illustrates how ICTs within these four broad categories have been used to support particular good–governance initiatives.

### Gathering and verifying information

Routine data collection is one of the more widely discussed use cases in the mHealth literature; it is well established that using mobile devices can improve data timeliness and quality [30]. Some governance initiatives have used the same or similar technologies to collect data for transparency or governance purposes. These initiatives include *organization–oriented* approaches, such as using mobile phones to collect data on governance practices or to independently verify routine government health statistics. For example, government health workers can use the USAID–funded GovScore app to report on institutional governance practices, such as in a project evaluating the formation of local health advisory committees [31]. The Performance Monitoring and Accountability 2020 project, sponsored by the Gates Foundation, uses the mobile app Open Data Kit to undertake public surveys about family planning and sanitation services in LMICs [32]. While this does not explicitly target governance challenges such as corruption, using data captured directly from civil society to verify routine health service statistics may enable external accountability in ways that are not possible for mHealth initiatives reliant on self–reporting by government health care providers.

Other information gathering approaches involve *engaging citizens* to crowdsource intelligence on health system performance, such as stock–outs, absenteeism, bribery or corruption. Rather than providing equipment (eg, smartphones) to a relatively small group of data gatherers, projects that solicit the participation of large groups of people are more likely to rely on technologies that people can access via the Internet or, particularly in LMIC settings, via the least expensive and most widely available mobile phones. In this vein, the social enterprise “Open Health Networks” enlisted members of the public to submit SMS reports of drug stock–outs and health worker discrimination against indigenous patients in Guatemala [33]. According to a press release by the Canadian Government’s International Development Research Centre, this data and subsequent community meetings resulted in seven municipalities increasing the amount of funding allotted to keep medicines in stock or provide fuel for ambulances [34]. One of the larger crowdsourcing for global health initiatives is UNICEF’s social platform U–Report, which originated in Uganda and now operates in several countries, with over a million registered users in Nigeria alone [35]. The large database of registered users enables U–Report to conduct massive polls. For example, data from 16 117 U–Report respondents was used in conjunction with traditional survey methods in a project assessing client satisfaction with services in Uganda’s public health facilities [36].

### Data aggregation and visualization

While data gathered digitally may simply be summarized in written reports and discussed in face–to–face meetings, we identified a second category of use cases related to *data aggregation and visualization*. Digital analysis tools can become necessary as data sets grow extremely large or when information management teams are understaffed. While digital analytics and visualization tools, such as dashboards and maps, are increasingly common throughout the health sector, their use in good governance interventions involves distinctive data sets (eg, on absenteeism) or civil society verification of government statistics. Thus such tools might enable comparison of drug stock–outs across multiple catchment areas or draw attention to “hot spots” of corruption that would be less obvious when viewing massive spreadsheets. A number of digital technologies integrate data gathering, analysis and visualization tools, either for organization–oriented governance or for citizen–centered approaches such as crowdsourced maps that can be used to negotiate change [37].

### Mobilization

A number of the articles and interventions we identified involved a *mobilization* component. Projects of this kind use ICTs to raise public awareness of corruption in order to generate political pressure for change or otherwise spur collective action aimed at reforming unethical or negligent health care practices. Such efforts reflect an important tenet of the contemporary transparency and accountability movement; that transparency alone is insufficient to drive greater government performance or accountability [16].

Some digital mobilization efforts unfold primarily online; for example through social media and blogging, eg, [4]. Others blend digital and “offline” approaches, such as pairing community meetings and poster campaigns with the information gathering and analytics tools discussed previously eg, [33]. Another common approach involves digital diary methods, where people experiencing a health issue are invited to document their own perspective and share photographs, audio or video with others eg, [38]. In some cases dialogue and mobilization are proactively cultivated by governments or through public private partnerships aiming to improve responsiveness or “feedback loops” among citizens and government actors [39,40]. In other cases, however, mobilization efforts are prioritized because government actors were initially unresponsive to citizen concerns. For example, in the 1990s over 300 000 indigenous Peruvian women and 20 000 indigenous men underwent forced sterilization through a state government “poverty reduction” campaign that was funded by international donors and initially supported by women’s rights organizations. With the aim of pressuring the government to acknowledge that the health policy was harmful, the Quipu Project uses mobile phones, radio, and an interactive documentary to communicate testimonies of those affected [41].

### Automation and auditing

Finally, *automation and auditing* may help to address inappropriate practices by taking processes or decisions out of the hands of individual health care personnel and intermediaries. For example, new algorithms can automatically detect “outlier” data sets that show signs of having been faked by an absentee worker rather than having emerged from a genuine patient encounter [42]. Electronic billing and e–cash registers may address informal payments or bribes [43] and disbursing money via mobile phones can address a major source of corruption, enabling ministries to ensure that full salaries go to the intended workers in a timely manner [32,44].

Digitizing processes can also increase auditability. For example, doubts regarding whether community health workers actually visit the homes of remote patients in their care may be addressed using biometric fingerprint technology to verify each patient visit [45]. Auditable databases of dispensed drugs are also being used to tackle the widespread problem of counterfeit medicines. This typically involves labeling all medicines with serial numbers so that purchasers who text message a unique code to an SMS hotline can verify that their product is registered eg, [46,47].

### Discussion

While a growing number of anecdotal reports suggest that digital interventions for good health sector governance hold promise, the relevant evidence is undeniably mixed. For every success, there have been outright failures, as is the case with conventional good governance interventions (for which there are more randomized trials) [16]. Numerous reports and expert interviews stressed that ICT for health governance interventions hinge on nuanced contextual factors and the challenge of linking transparency and action.

Our analysis revealed fifteen unique use cases of ICT for good governance, clustering into four conceptual categories associated with better information for transparency, usable data for decision making, citizen mobilization for accountability and process automation for fraud prevention. While most of these use cases targeted government–sponsored services, some extended to the private sector, such as those aimed at combatting drug counterfeiting. Since the private health care sector is dominant in many low– and middle–income countries we anticipate seeing more ICT for good–governance focused on these settings in the future, mindful of the role of government in ensuring that these are effectively regulated.

It should be noted that, while we organized our findings around a collection of generic digital tools, in practice there is a tendency to mix and match two or more of these as components of integrated interventions that enable governance processes. Such integrated governance interventions aim to strengthen citizen–government “feedback loops” [42,48], or complete an “action cycle” [16], using data to drive performance improvement or responsiveness in government services through particular mechanisms. For example, the My Voice project in Nigeria enabled citizens visiting health facilities to send feedback via SMS; the feedback was visualized for government leaders through an online dashboard, and these leaders then completed a feedback loop by using citizen–generated data in their routine review meetings regarding the performance of specific health facilities [42]. This reflects a growing acknowledgment that transparency alone (eg, having an Open Government data portal) is insufficient to influence governance [16,49]. To put this in other terms, it should not be assumed that digital technologies or eGovernment platforms will deliver good governance–related benefits unless they explicitly address specific and measurable concerns with performance or facilitate concrete mechanisms of responsive governance.

Factors limiting the effectiveness of digital good governance inventions in developing countries include lower rates of Internet access and mobile phone ownership among women and vulnerable groups [42,50,51]. Poor local network access or smartphone penetration could further marginalize people who already have less influence in governance. Understanding local patterns of technology use is therefore vital when determining which components of good governance interventions should be digitized and which are better left offline. The extent to which governments are responsive to public concerns also has implications for which approaches are likely to succeed [16]; indeed complaining about the government can be dangerous for citizens in some countries.

Finally, the preponderance of reports and expert interviews indicated that digital good governance interventions in health care are deeply complex. Their outcomes hinge on distinctive political factors in addition to the myriad organizational and sociotechnical dynamics that shape digital health innovation generally [52]. The value of human–centered design (HCD) in addressing such complexities is increasingly recognized in reports on ICT for health governance [31,33,42] as well as broader consensus statements, such as the widely ratified Principles for Digital Development [53]. HCD principles and practices include grounding the project in insights from fieldwork undertaken in the context of use, involving end–users in the design process, and iteratively adapting to feedback and experiential insights. Such approaches share important conceptual and philosophical links with transparency and accountability interventions, the former influenced by Scandinavia’s participatory design movement [54], and the latter by the broader participatory global development community [16]. In both communities, participatory approaches reflect practical priorities as well as the democratic view that ordinary people should have a say in matters that affect them. While participatory co–design will not guarantee the effectiveness of digital good–governance interventions, it offers practical resources for dealing with complex design situations and merits further attention, given its links with participatory approaches to governance.

### Limitations

In keeping with our research aims and with methodological guidance for scoping reviews [24,25], our search and study inclusion process was broader than would be typical for a fully systematic review but also more limited, in terms of databases, keywords and the absence of critical appraisal. As already noted, scoping reviews are best understood as *hypothesis generating* activities rather than *hypothesis testing* endeavors, and are useful for mapping emerging areas with scattered literatures. In this case, the scoping process proved extremely valuable in helping us to refine our research focus, align with appropriate theoretical literature and converge different types of evidence to better understand this evolving interdisciplinary area. In addition to undertaking all of the research stages recommended for scoping reviews, we conducted two stages that are typically considered optional: a landscape scan of implementation activities and consultation with experts and practitioners. We recommend that future systematic reviewers wishing to build on this work use a wider range of databases, including those specializing in research from LMIC, as well as more exhaustive search methods.

## CONCLUSIONS AND RECOMMENDATIONS

Recent years have seen a rapid growth in the number and scale of ICT for health governance projects in LMICs. This trend seems likely to continue, with advances in digital infrastructure and Sustainable Development Goal 16 drawing further attention to strong institutions, public participation and combatting corruption [2].

Among the numerous reports discussing ICTs and good governance that we examined, we observed a tendency to emphasize data or transparency alone, with the implicit assumption that improvements in the quality or equity of health services would inevitably follow. However, the evidence suggests that the link between ICTs, transparency and improved performance should not be taken for granted, echoing observations from our recent scoping review on the use of social media for e–government [55]. In order to be effective ICT enabled good governance interventions should address practical, specific and measurable concerns with health sector performance, with the long–term aim of improving the lives of citizens. We recommend that policymakers, sponsors and implementers of these initiatives prioritize the proactive use of data to drive reform, establishing citizen–government feedback loops and mechanisms of accountability, with a view to completing “action cycles” rather than settling for transparency or better information alone [16].

Further research is required to strengthen the theoretical models underpinning these approaches and articulate their pathways to impact, while empirical studies are needed to evaluate their outcomes and understand factors mediating their adoption or effectiveness. Human–centered and participatory approaches to intervention design also merit greater attention, not only as a practical means of dealing with local complexities, but also for their links with participatory approaches to governance.

To our knowledge, this is the first formal scoping review to have examined the literature on ICT for good governance interventions in the context of LMIC health care systems. These interventions show great promise for improving transparency, accountability and public participation, thereby facilitating ethical, responsible and equitable health care. However, existing evidence of their use and effectiveness is mixed and successes appear highly context–dependent. As well as adding to the wider multi–sector literature on this topic, we hope our observations provide useful insights for policymakers, practitioners, developers and sponsors considering new projects in this area.

## References

[1] Jain A, Nundy S, Abbasi K (2014). Corruption: medicine’s dirty open secret.. BMJ.

[2] Sustainable Development Goal 16. Available: http://www.un.org/sustainabledevelopment/peace-justice. Accessed: 6 November 2015.

[3] Sheng YK. What is good governance. United Nations Economic and Social Commission for Asia and the Pacific. Available: http://www.unescap.org/sites/default/files/good-governance.pdf. Accessed: 21 May 2016.

[4] Bjorkman M Svensson J Power to the people: evidence from a randomized field experiment of a community–based monitoring project in Uganda. Quarterly Journal of Economics. 2009:124:735-69.

[5] I Paid a Bribe. Available: http://www.ipaidabribe.com. Accessed: 20 September 2015.

[6] All U–Report Members. Available: http://www.ureport.in. Accessed: 20 November 2015.

[7] Palvia SCJ, Sharma SS. E–government and e–governance: definitions/domain framework and status around the world. 2007. http://www.iceg.net/2007/books/1/1_369.pdf. Accessed: 21 March 2016.

[8] Friedman CP, Wong AK, Blumenthal D (2010). Achieving a nationwide learning health system.. Sci Transl Med.

[9] Scally G, Donaldson LJ (1998). Clinical governance and the drive for quality improvement in the new NHS in England.. BMJ.

[10] Vanu Som C. Clinical governance: a fresh look at its definition. Clinical Governance. 2004;9:87-90.

[11] Gaventa J. Towards participatory governance: assessing the transformative possibilities. In: Hickey S, Mohan G, eds. Participation: From tyranny to transformation. London: Zed Books, 2004. p. 25-41.

[12] Dodgson R, Lee K, Drager N. Global health governance: a conceptual review. Centre on Global Change & Health, London School of Hygiene & Tropical Medicine. 2002. Available: http://apps.who.int/iris/bitstream/10665/68934/1/a85727_eng.pdf. Accessed: 2 June 2016.

[13] Thomas C, Weber M (2004). The politics of global health governance: whatever happened to “health for all by the year 2000”?. Glob Gov.

[14] Mackey TK, Liang BA (2013). A United Nations global health panel for global health governance.. Soc Sci Med.

[15] Frenk J, Moon S (2013). Governance challenges in global health.. N Engl J Med.

[16] Kosack S, Fung A (2014). Does transparency improve governance?. Annu Rev Polit Sci.

[17] Olken B, Pande R, Dhaliwal I, Dragusanu R, Marshall C. Governance Review Paper: J–PAL Governance Initiative Cambridge, MA: Abdul Latif Jameel Poverty Action Lab., 2011.

[18] Joshi A, Houtzager PP (2012). Widgets or watchdogs? Conceptual explorations in social accountability.. Public Manage Rev.

[19] World Bank Staff. World development report 2004: making services work for poor people. World Bank Publications, 2003.

[20] Bennett WL, Howard PN. Evolving public–private partnerships: a new model for e–Government and e–Citizens. Partnerships for Technology Access. 2008. Available: http://download.microsoft.com/download/0/2/2/0222c02b-ea6f-4d53-a6c2-0587d096a121/evolving_public-private_partnerships_lores.pdf. Accessed: 3 March 2016.

[21] Estevez E, Janowski T (2013). Electronic governance for sustainable development: conceptual framework and state of research.. Government Information Quarterly.

[22] Hovenga EJ, Grain H, editors. Health information governance in a digital environment. Ios Press, 2013.

[23] Holeman I, Cookson TP, Pagliari C. Digital technology for health sector governance. Leadership, Management and Governance (LMG) Project. USAID, 2016. 10.7189/jogh.06.020408 PMC5017033 27648255

[24] Armstrong R, Hall BJ, Doyle J, Waters E (2011). ‘Scoping the scope’ of a Cochrane review.. J Public Health (Oxf)..

[25] Arksey H, O'Malley L (2005). Scoping studies: towards a methodological framework.. Int J Soc Res Methodol.

[26] Holeman I, Cookson TP, Pagliari C. Digital technology for health sector governance. Leadership, Management and Governance (LMG) Project. USAID, 2016. 10.7189/jogh.06.020408 PMC5017033 27648255

[27] Ahmed T, Lucas H, Khan AS, Islam R, Bhuiya A, Iqbal M (2014). eHealth and mHealth initiatives in Bangladesh: a scoping study.. BMC Health Serv Res.

[28] Philbrick W. mHealth and MNCH: state of the evidence. Trends, Gaps, Stakeholder Needs, and Opportunities for Future Research on the Use of Mobile Technology to Improve Maternal, Newborn, and Child Health. Washington: UN Foundation, 2015. Available: http://www.mhealthknowledge.org/sites/default/files/15_un_007_evidencegapreport_digital_aaa.pdf. Accessed: 6 November 2016.

[29] Levac D, Colquhoun H, O’Brien KK (2010). Scoping studies: advancing the methodology.. Implement Sci.

[30] Blaya JA, Fraser HSF, Holt B (2010). E–health technologies show promise in developing countries.. Health Aff (Millwood).

[31] Wangui A, Macharia M. Medic Mobile Field Research on Leadership, Management and Governance Feedback and Collection Tools. Nairobi: iHub UX Lab, 2015.

[32] Gichangi P. Detailed indicator report: Kenya 2014. Performance Monitoring and Accountability 2020 (PMA2020). 2014. Available: http://www.pma2020.org/sites/default/files/PMAKE-DIR-2015.04.27.pdf. Accessed: 24 September 2015.

[33] Kahane M, Prachanronarong P. Open Health Networks Implementation Guide V.1. Open Health Networks. 2015. Available: http://cegss.osf.parsons.edu/#deliverables. Accessed: 10 March 2016.

[34] IDRC. Forum unveils innovative approaches to secure health care rights for Guatemala’s indigenous communities. 1 May 2015. Available: http://www.idrc.ca/EN/Themes/Health/Pages/NewsDetails.aspx?NewsID=698. Accessed: 10 March 2016.

[35] UNICEF. UNICEF’s U–Report hits 1 million users. 16 July 2015. Available: http://www.unicef.org/media/media_82583.html Accessed: 24 June 2016.

[36] Higenya E, Ekwaro G, Seru M. Client satisfaction with services in Uganda’s public health facilities. Medicines Transparency Alliance of Uganda. 2014. Available: http://apps.who.int/medicinedocs/en/d/Js21905en/. Accessed: 6 November 2015.

[37] Bott M, Young G (2012). The role of crowdsourcing for better governance in international development. Praxis. The Fletcher Journal of Human Security..

[38] Green E, Kloos B (2009). Facilitating youth participation in a context of forced migration: a photovoice project in northern Uganda.. J Refug Stud.

[39] Voices for good governance. ENCISS: For Rights and Voice. 2014. Available: http://www.enciss-sl.org/node/537. Accessed: 10 March 2016.

[40] The World Bank and Reboot. Enabling citizen–driven improvement of public services: leveraging technology to strengthen accountability in Nigerian healthcare. The World Bank. 2015. Available: http://reboot.org/wordpress/wp-content/uploads/2015/03/Enabling-Citizen-Driven-Improvement-of-Public-Services_2015.pdf. Accessed: 6 November 2015.

[41] Tucker K, Brown M. The Quipu project: Participatory story–telling can help rebuild community in post–authoritarian societies. PolicyBristol. Available: http://www.bristol.ac.uk/media-library/sites/policybristol/migrated/documents/quipuproject.pdf. Accessed: 7 March 2016.

[42] McCarthy T, DeRenzi B, Blumenstock J, Brunskill E. Towards operationalizing outlier detection in community health programs. In: Proceedings of the Sixth International Conference on Information and Communications Technologies and Development: Notes–Volume 2; 2013 Dec 7; p. 88-91.

[43] Vian T. Anti–corruption in the health sector: reducing vulnerabilities to corruption in user fee systems. U4 Brief. 2006;3.

[44] Blumenstock JE, Callen M, Ghani T, Koepke L. Promises and pitfalls of mobile money in Afghanistan: evidence from a randomized control trial. In: ICTD. 2015 May 15; p. 15.

[45] JPAL. Poverty Action Lab. Using the DOTS model to combat tuberculosis in India. Available: http://www.povertyactionlab.org/evaluation/using-dots-model-combat-tuberculosis-india. Accessed: 6 November 2015.

[46] mPedigree Goldkeys. Available: http://goldkeys.org. Accessed: 6 September 2015.

[47] Sproxil. Available: http://sproxil.com/sms-verification.html. Accessed: 23 January 2015.

[48] Voices for good governance. ENCISS: For Rights and Voice. 2014. Available: http://www.enciss-sl.org/node/537. Accessed: 10 March 2016.

[49] Democratic Audit UK. Unless we change the way we think about transparency, open data is unlikely to have a significant political impact at the local level. 2015. Available: http://www.democraticaudit.com/?p=13606. Accessed: 6 April 2016.

[50] United Nations. Chapter 6: Bridging The Digital Divide, United Nations e–government survey 2014. New York: Department of Economic and Social Affairs, 2014.

[51] ITU. ICT for Improving Information and Accountability for Women's and Children's Health. July 2013. Available: https://www.itu.int/en/ITU-D/ICT-Applications/Documents/CoIA%20Background%20ICT4RMNCH.pdf. Accessed: 10 April 2016.

[52] Holeman I, Evans J, Kane D, Grant L, Pagliari C, Weller D (2014). Mobile health for cancer in low to middle income countries: priorities for research and development.. Eur J Cancer Care (Engl).

[53] Principles for Digital Development. Available: http://digitalprinciples.org. Accessed: 3 March 2016.

[54] Bannon LJ, Ehn P. Design: design matters in participatory design. In: Simonsen J, Robertsen T. (eds) Routledge international handbook of participatory design. New York: Routledge, 2012. p.37-63.

[55] Franco M, Tursunbayeva A, Pagliari C (2016). Social media for e–Government in the public health sector: protocol for a systematic review.. JMIR Res Protoc..

