# Supplementary material for: Digital technology for health sector governance in low and middle income countries: a scoping review
Source: J Glob Health. 2016 Sep 7;6(2):020408. doi: 10.7189/jogh.06.020408 (PMC5017033; doi:10.7189/jogh.06.020408)
Supplement: Online Supplementary Document [file jogh-06-020408-s001.pdf]

Online Supplementary Document

Holeman et al. Digital technology for health sector governance in low and middle income countries: a scoping review

J Glob Health 2016;6:020408

Appendix S1: Peer Reviewed Research

The following list includes 17 peer-reviewed articles that met this review's inclusion criteria, discovered in databases of academic research, through reference lists, and through consultation with industry experts and online communities.

Birnbaum, B. et al. (2012). Automated quality control for mobile data collection. In Proceedings of the 2nd ACM Symposium on Computing for Development. ACM, pp. 1–10.

Birnbaum, B., Borriello, G., Flaxman, A. D., DeRenzi, B., & Karlin, A. R. (2013). Using behavioral data to identify interviewer fabrication in surveys. In Proceedings of the SIGCHI Conference on Human Factors in Computing Systems (pp. 2911-2920). ACM.

Boss, S. (2015). Getting Creative About Corruption. Stanford Social Innovation Review. Summer, 8-12.

Bott, M., Young, G. (2012). The Role of Crowdsourcing for Better Governance in International Development. Praxis: The Fletcher Journal of Human Security, 27 (1), 47-70.

de Lange, N. and Mitchell, C. (2012). Community Health Workers Working the Digital Archive: A Case for Looking at Participatory Archiving in Studying Stigma in the Context of HIV and AIDS. Sociological Research Online, 17 (1), 7.

Erikson, Susan L. (2012). Global Health Business: The Production and Performativity of Statistics in Sierra Leone and Germany. Medical Anthropology: Cross-Cultural Studies in Health and Illness, 31:4, 367-384.

Estuar, M. R., Batangan, D., Coronel, A., Amarra, A. C., & Castro, F. (2013). Bottom Up Approach and Devolved Design of a Health Information System: eHealth TABLET. In Brain and Health Informatics (pp. 210-217). Springer International Publishing.

Green, E., & Kloos, B. (2009). Facilitating youth participation in a context of forced migration: a Photovoice project in northern Uganda. Journal of Refugee Studies, 22(4), 460-482.

Islam, M. S. (2015). The Impact of Transparency on Quality of Health Service Delivery in Bangladesh: Findings of a Field Survey of Rural and Urban Health Service Organisations. Journal of Nursing and Health Care (JNHC), 2(1).

Islam, M. S. (2015). Introducing modern technology to promote transparency in health services. International journal of health care quality assurance, 28(6), 611-620.

Maru, D. S. R., Sharma, A., Andrews, J., Basu, S., Thapa, J., Oza, S., ... & Schwarz, R. (2009). Global health delivery 2.0: using open-access technologies for transparency and operations research. PLoS medicine, 6(12), 1329.

McCarthy, T., DeRenzi, B., Blumenstock, J., & Brunskill, E. (2013, December). Towards operationalizing outlier detection in community health programs. In Proceedings of the Sixth International Conference on Information and Communications Technologies and Development: Notes-Volume 2 (pp. 88-91). ACM.

Mikkelsen-Lopez, I., Shango, W., Barrington, J., Ziegler, R., & Smith, T. (2014). The challenge to avoid anti-malarial medicine stock-outs in an era of funding partners: the case of Tanzania. Malaria journal, 13(1), 181.

Kluge, E. (2008). Ethical aspects of future health care: globalisation of markets and differentiation of societies-ethical challenges. Studies in health technology and informatics, 134, 77.

Roess, A., Gurman, T., Ghoshal, S., & Mookherji, S. (2014). Reflections on the Potential of mHealth to Strengthen Health Systems in Low-and Middle-Income Countries. Journal of health communication, 19(8), 871-875.

Weimann, E., & Stuttaford, M. C. (2014). Consumers' perspectives on National Health Insurance in South Africa: using a mobile health approach. JMIR mHealth and uHealth, 2(4).

Wójcik, O. P., Brownstein, J. S., Chunara, R., & Johansson, M. A. (2014). Public health for the people: participatory infectious disease surveillance in the digital age. Emerging themes in epidemiology, 11(1), 7.

Appendix S2: Reports and Other Grey Literature

This list includes the 17 published reports that met all of this study's inclusion criteria, discovered through reference lists, Google searches and through consultation with industry experts and online communities.

Blaschke, S. and Grépin, K. (2014). Strengthening accountability chains for maternal, newborn and child health in Uganda – UNICEF's mTrac", World Health Organization.

Croke K, Dabalen A, Demombynes G, Giugale M, Hoogeveen J. Collecting High-Frequency Data Using Mobile Phones: Do Timely Data Lead to Accountability?.

Fahmy, A., Abdelmonem, A., Hamdy, E., Badr, A. (2014). Sexual Harassment in Greater Cairo: Effectiveness of Crowdsourced Data. HarassMap in collaboration with Youth and Development Consultancy Institute (Etijah).

Gichangi, P. (2015). Detailed Indicator Report: Kenya 2014. Performance Monitoring and Accountability 2020 (PMA2020). Baltimore, MD.

Higenya, E., Ekwaro, G., Seru, M. (2014). Client Satisfaction with Services in Uganda's Public Health Facilities. Medicines Transparency Alliance of Uganda.

Kahane, M., Prachanronarong, P. Open Health Networks Implementation Guide V.1 [Internet]. Open Health Networks; 2015 [cited 2016 March 10]. Available from: http://cegss.osf.parsons.edu/#deliverables.

Kickbush, I., Gleicher, D. (2012). Governance for Health in the 21st Century. World Health Organization.

Otupiri, E., 2013. Detailed Indicator Report: Ghana. Performance Monitoring and Accountability 2020 (PMA2020). Baltimore, MD.

Leah, F., Boots, M. Barries and Solutions in using M4D: Connecting Directly to Citizens for Scalable Impact. Kumasi, Ghana: Voto Mobile; 2013.

Makumbi, F. and Kibira, SP. (2015). Detailed Indicator Report: Uganda 2014. Performance Monitoring and Accountability 2020 (PMA2020). Baltimore, MD.

Rice, James A., Shukla, Mahesh, Johnson Lassner, Karen et al. (2015). Leaders Who Govern. Management Sciences for Health. Arlington, VA.

Renata, Avila, Feigenblatt, Hazel, Heacock, Rebekah, Heller, Nathaniel (2010). Global mapping of technology for transparency and accountability. Open Society Foundation. London, UK.

Reboot (2015). "Enabling citizen-driven improvement of public services: Leveraging technology to strengthen accountability in Nigerian healthcare" Reboot and World Bank

Shiferaw, S. and Seme, A. (2014). Detailed Indicator Report: Ethiopia. Performance Monitoring and Accountability 2020 (PMA2020). Baltimore, MD.

Tucker, K. and Brown, M. (2014) The Quipu project: Participatory story-telling can help rebuild community in post-authoritarian societies.

Voices for good governance [Internet]. Place unknown: ENCISS: For Rights and Voice; 2014 [cited 2016 March 10]. Available from: http://www.enciss-sl.org/node/537

Wangui, A., Macharia, M. Medic Mobile Field Research on Leadership, Management and Governance Feedback and Collection Tools. Nairobi: iHub UX Lab; 2015.
